# Infantile-onset pompe disease: a case report emphasizing the role of genetic counseling and prenatal testing

**DOI:** 10.1186/s12887-024-04690-6

**Published:** 2024-03-18

**Authors:** Yasaman Alizadeh, Hossein Saidi, Vahid Saeedi, Leila Kamalzadeh

**Affiliations:** 1 Pediatric Endocrinology and Metabolism Department, School of Medicine, Iran University of Medical Sciences, Tehran, Iran; 2 Pediatric Critical Care Unit, Rasool Akram Medical Complex Clinical Research Development Center (RCRDC), School of Medicine, Iran University of Medical Sciences, Tehran, Iran; 3 Department of Psychiatry, Rasool Akram Medical Complex Clinical Research Development Center (RCRDC), School of Medicine, Iran University of Medical Sciences, Tehran, Iran

**Keywords:** Pompe disease, Glycogen Storage Disease type II, Alpha-glucosidases, Family Planning services, Genetic counselling, Prenatal diagnosis

## Abstract

**Background:**

Pompe disease, classified as glycogen storage disease type II, arises from a deficiency in the acid alpha-glucosidase (GAA) enzyme, leading to glycogen accumulation in multiple tissues. The unique correlation between genotype and enzyme activity is a key feature. This case highlights an infantile-onset form, emphasizing genetic counseling and prenatal testing importance.

**Case Presentation:**

An 18-week-old infant with respiratory distress, cyanosis, and fever was admitted. Born healthy, her sibling died from Pompe disease. She presented with cardiomegaly, hypotonia, and absent reflexes. Diagnosis was confirmed by significantly reduced GAA activity. Despite treatment initiation, the patient succumbed to cardiac arrest.

**Conclusions:**

The case underscores genetic counseling’s role, offering insights into prenatal testing advancements, antenatal diagnosis through echocardiography, and the significance of early intervention, particularly in infantile-onset Pompe disease.

**Synopsis:**

Genetic risk assessment and prenatal testing are crucial for families with a history of Pompe disease to improve early diagnosis and management outcomes.

## Introduction

Pompe disease, classified as glycogen storage disease type II, is an infrequent metabolic disorder resulting from a deficiency of the lysosomal acid alpha-glucosidase (GAA) enzyme. The enzymatic deficiency results in glycogen accumulation within the lysosome and cytoplasm across various tissues, with heart and skeletal muscle being the most severely affected [1]. The unique correlation between genotype and enzyme activity is a key feature, as it is observed that patients with the infantile-onset phenotype exhibit an absence or minimal level of enzyme activity. In contrast, late-onset subtypes exhibit varying degrees of enzyme reduction [2].

Infantile-onset Pompe disease (IOPD) is the most severe form and is characterized by hypertrophic cardiomyopathy detectable during fetal development and significant generalized hypotonia in early infancy. Clinical manifestations include cardiomegaly, respiratory insufficiency, poor muscle tone, feeding difficulties, and failure to thrive, typically appearing around four months of age [3, 4]. The median survival duration for IOPD patients without therapy is fewer than two years, owing to cardiorespiratory failure. Late-onset forms do not typically present with cardiac manifestations [5].

GAA deficiency follows an autosomal-recessive inheritance pattern, where both copies of the responsible gene must be affected for the disorder to manifest. The condition displays considerable allelic heterogeneity, with more than 634 reported variants causing the disease. In families with a Pompe disease diagnosis, relatives are at risk of being carriers or could be affected but presymptomatic (in the late-onset type). Providing genetic counseling to young adults affected or at risk of being carriers is essential [6]. Prenatal testing, either by measuring GAA enzyme activity or, in cases with known familial mutations, molecular testing, provides avenues for early diagnosis [3]. Enzyme replacement therapy (ERT) has shown promise in improving Pompe disease patients’ survival rates and clinical outcomes. However, its efficacy depends on initiating treatment before irreversible muscle damage occurs. This report presents a case of infantile-onset Pompe disease, highlighting the significance of genetic counseling and prenatal testing to prevent recurrence in high-risk families [1, 7].

## Case presentation

An 18-week-old female infant weighing 5950 g was admitted to the hospital due to respiratory distress, cough, cyanosis, and fever that had persisted for six days. The infant was born at full term through an elective repeat cesarean section, with a birth weight of 3195 g and no congenital anomalies or delivery complications. She attained normal developmental milestones until two months of age, when decreased activity was observed. Both parents were in good health and unrelated. This child was their third offspring. Their first child was healthy, but their second child died at five months of age due to Pompe disease.

The infant’s vital signs upon admission were as follows: a temperature of 38.3 °C, a heart rate of 147 beats/min, a respiratory rate of 47 breaths/min, a blood pressure of 90/60 mmHg, and an oxygen saturation of 92% on room air. Clinical examination revealed lethargy, pallor, and cyanosis. Physical characteristics included a flattened nasal bridge and a slight macroglossia. Mid-systolic murmurs were detected in cardiac auscultation. Both lungs had rales on examination. The abdomen was non-tender and soft to the touch with no signs of organ enlargement. A neurological examination revealed generalized hypotonia and absent deep tendon reflexes in all four limbs. The infant had suboptimal eye contact and poor head control. The patient was admitted to the pediatric intensive care unit.

Initial laboratory investigations yielded the following results (Table 1):

**Table 1:** Laboratory tests performed on the patient. Results outside the reference interval are marked in bold

Parameter	Value	Reference Range
Hemoglobin	10.8 g/dL	9.5–13 g/dL
MCV	84.9 fL	80–95 fL
White blood cell count	**15.1 × 10^9/L**	5–10 × 10^9/L
Platelet count	429 × 10^9/L	150–450 × 10^9/L
Sodium	**132 mmol/L**	135–145 mmol/L
Potassium	4.4 mmol/L	3.5-5 mmol/L
Blood urea nitrogen	8.6 mg/dL	5–18 mg/dL
Creatinine	0.51 mg/dL	0.3–0.7 mg/dL
Glucose	**123 mg/dL**	70–110 mg/dL
Triglyceride	111 mg/dL	60–160 mg/dL
Calcium	10.1 mg/dL	8.5–10.5 mg/dL
Phosphorus	4.7 mg/dL	4–7 mg/dL
Magnesium	2.2 mg/dL	1.7–2.4 mg/dL
Albumin	**2.5 g/dL**	3.5–5.5 g/dL
Alanine aminotransferase	**74 U/L**	< 40 U/L
Aspartate aminotransferase	**163 U/L**	< 40 U/L
Alkaline phosphatase	**563 U/L**	< 350 U/L
Lactate dehydrogenase	**1600 U/L**	200–500 U/L
Creatine kinase	498 U/L	24–195 U/L
Brain natriuretic peptide	**11,800 pg/mL**	< 100 pg/mL
Troponin I	**0.22**	< 0.04
C-reactive protein	**20 mg/L**	< 5 mg/L
Erythrocyte sedimentation rate	8 mm/hr	≤ 10 mm/hr
Arterial blood gas	pH 7.40	pH 7.35–7.45
	pCO2 35.5 mmHg	pCO2 35–45 mmHg
	**pO2 43.7 mmHg**	pO2 > 80 mmHg
	**HCO3 21.2 mmol/L**	HCO3 22–26 mmol/L
	**O2 saturation 79.8%**	O2 saturation > 95%

**Fig. 1:**
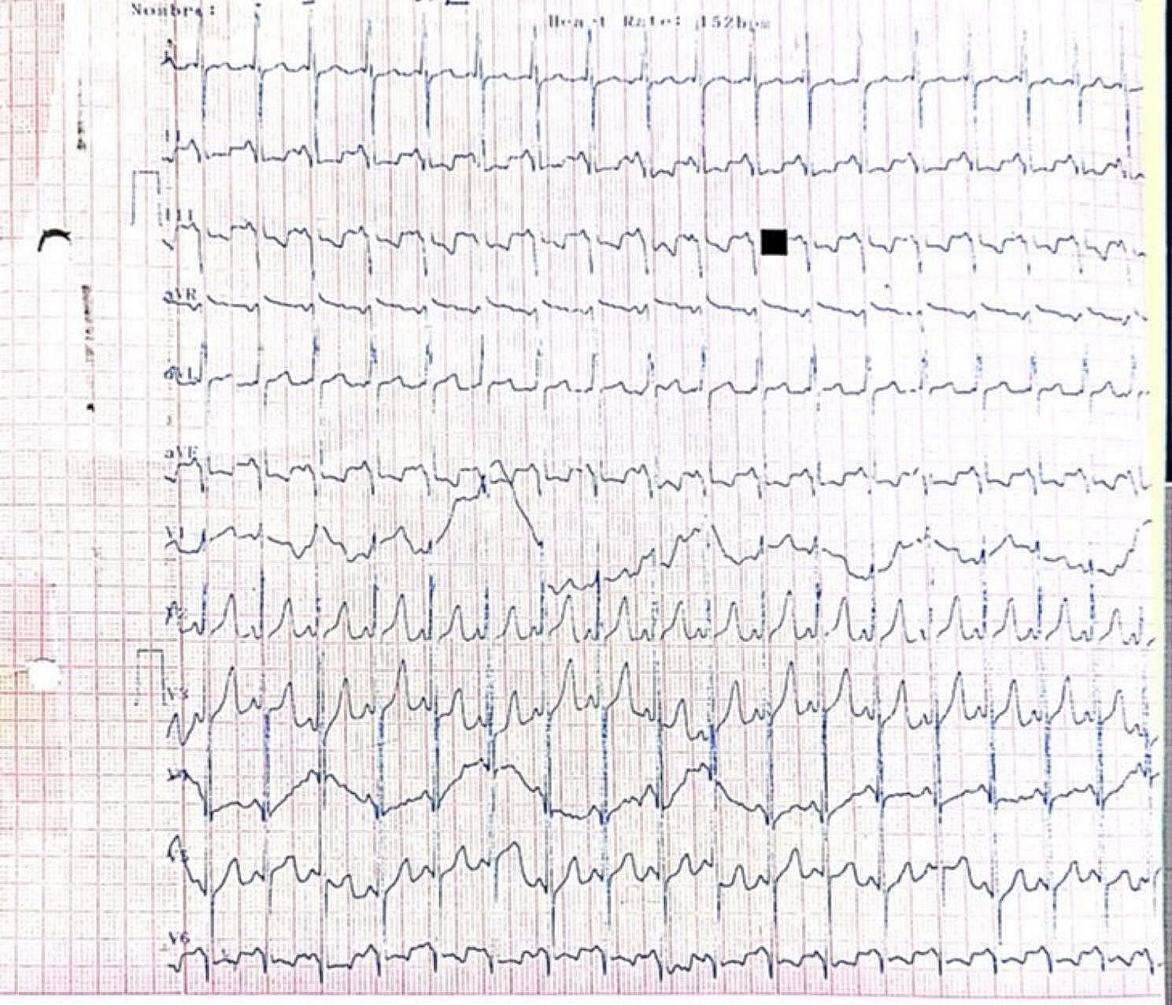
Admission electrocardiogram shows sinus tachycardia, short PR interval, ahigh-voltage R in left precordial leads, and strain pattern in V6 suggestive of left ventricular (LV) hypertrophy

The results of the urinalysis, urine culture, and blood cultures returned negative. Positive results were obtained from the tracheal secretion cultures for the presence of Acinetobacter. The COVID-19 polymerase chain reaction (PCR) test, as well as tests for Influenza A and B, were negative. The electrocardiogram showed sinus tachycardia, short PR interval, ahigh-voltage R in left precordial leads, and strain pattern in V6 suggestive of left ventricular (LV) hypertrophy (Fig. 1).

**Fig. 2:**
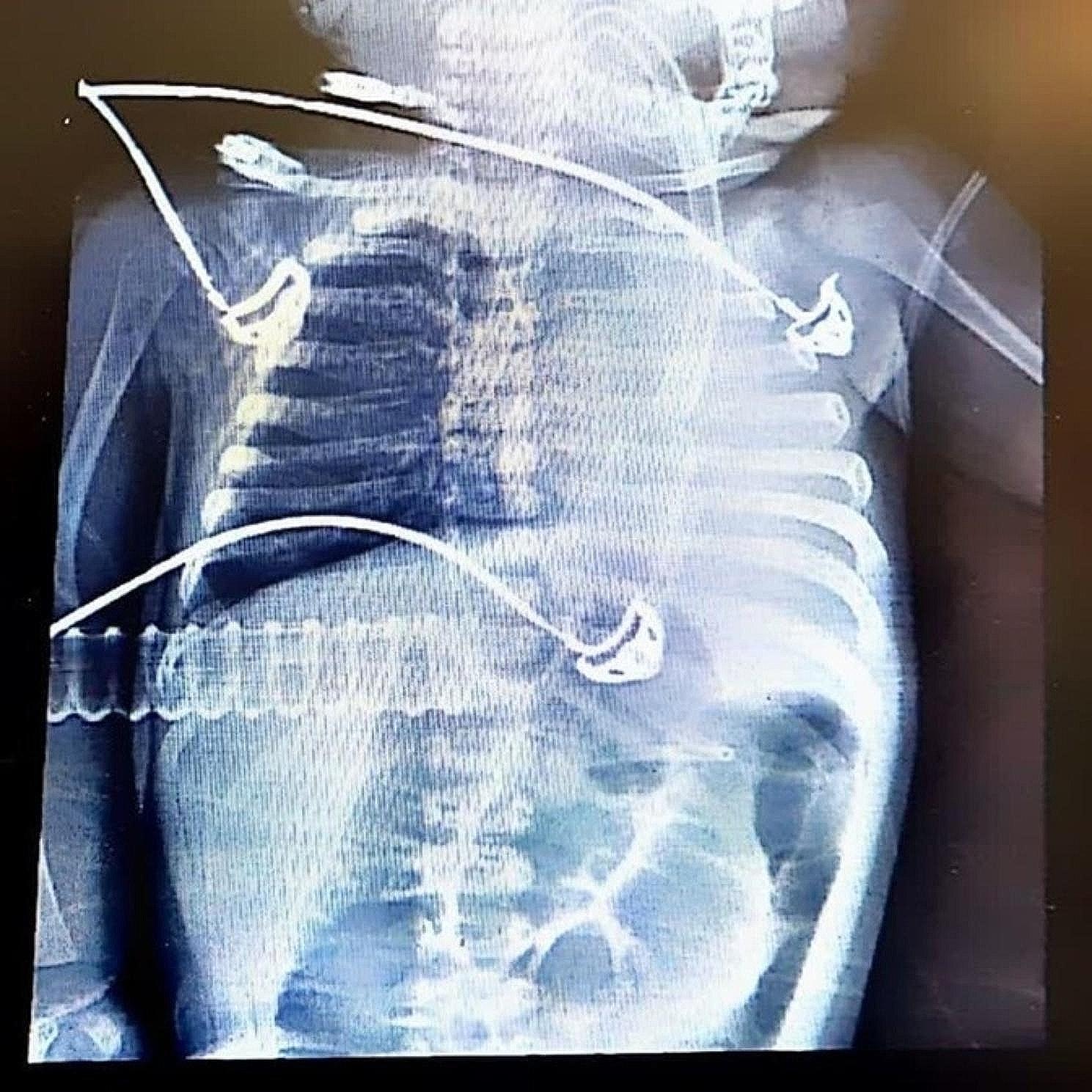
Admission Chest X-ray demonstrating cardiomegaly

Echocardiography demonstrated biventricular severe hypertrophy, significant LV systolic-diastolic dysfunction, and high LV filling pressure. The interventricular septum measured 8 mm. The LV ejection fraction was 30–35%, and mild mitral valve and tricuspid valve regurgitations were present. Chest X-ray revealed cardiomegaly with an increased cardiothoracic ratio (Fig. 2).

Pompe disease was suspected based on the patient’s clinical features and positive family history. Dried blood spot testing for Alpha-1,4 glucosidase (GAA) activity using tandem mass spectrometry confirmed the diagnosis of Pompe disease, showing significantly reduced enzymatic activity of 0.4 µmol/L/h (reference range for normal individuals: > 2.0 µmol/L/h). Genetic testing was not performed due to financial constraints the patient’s family faced. The patient received non-invasive ventilation with a nasal mask and continuous positive airway pressure of 5 cm H2O. Enteral nutrition was administered through a nasogastric tube using a high-calorie formula. Metoprolol was initiated at 0.5 mg/kg/day to suppress myocardial remodeling and reduce oxygen consumption. Antibiotics were prescribed for suspected pneumonia, and antipyretics were given to manage fever. The patient was referred for ERT with recombinant human GAA (rhGAA), but she succumbed to cardiac arrest before treatment initiation.

## Discussion

### Genetic counseling and family planning

This case report describes an 18-week-old female infant with IOPD who died before receiving ERT. Despite having a child previously diagnosed with Pompe disease, the family in this case did not pursue genetic counseling and prenatal testing for their subsequent pregnancy. This decision was influenced by multiple factors. Financial constraints posed significant barriers, given the high costs of genetic services, particularly in contexts where insurance coverage is limited or absent. The health of their first child, unaffected by the disease, may have inadvertently provided the parents with a false sense of security about the genetic risks for future children. Additionally, sociocultural beliefs could have played a role, as some families perceive discussions on genetic risks or prenatal diagnoses as stigmatizing. The intricate interplay of these factors highlights the challenges families face when confronted with genetic decisions [8, 9]. This underscores the importance of facilitating access to genetic counseling for Pompe-affected families. Enhanced healthcare provider awareness, prioritization in public health initiatives, incorporation into newborn screenings, and cultural sensitivity can collaboratively address these challenges and mitigate similar future outcomes [10].

### Advancements in prenatal testing

Traditional prenatal diagnosis of Pompe disease involved measuring GAA enzyme activity in chorionic villus tissue or amniocytes [5]. Advanced molecular genetic techniques now offer enhanced accuracy in prenatal diagnoses via complete gene sequencing and targeted mutation analysis. However, their efficacy hinges on the known pathogenic variants within the family. Absent this knowledge, molecular testing risks producing false negative results [11]. In this case, molecular genetic testing was not conducted for the infant or the previously affected sibling, primarily due to financial barriers. This situation underscores the significant challenges in accessing advanced prenatal testing in contexts where such services are expensive, despite their potential to enhance diagnostic accuracy and enable timely interventions [6].

### Antenatal diagnosis through echocardiography

In cases where prenatal enzyme activity or genetic testing is not feasible, fetal echocardiography in the third trimester has demonstrated its utility in diagnosing Pompe disease before birth. Cardiac features, such as hypertrophic cardiomyopathy, may serve as indicators, aiding in early recognition and management [12, 13]. However, the sensitivity of fetal echocardiography in the third trimester for identifying major congenital heart defects varies widely in literature, ranging from 55% to over 90% [14]. For detecting cardiac abnormalities associated with metabolic disorders such as Pompe disease, this sensitivity may be lower due to the subtleness of symptoms. Some cases could be missed or misinterpreted, especially if the examiner does not have a high index of suspicion based on other clinical parameters or family history [15].

### Newborn screening: an avenue for early detection

Worldwide, numerous nations have incorporated newborn screening (NBS) for Pompe disease due to its critical importance in early identification and intervention. The screening for GAA deficiency is generally executed using a stepwise approach, often starting with an enzymatic assay followed by molecular genetic testing. This systematic approach enables the initiation of ERT often before significant clinical manifestations emerge [16]. By the year 2023, 37 out of the 53 states and territories in the US had included Pompe disease in their NBS protocols, highlighting the crucial role of early detection in disease management [17]. In Iran, while many major cities have implemented NBS for Pompe disease, there remains a disparity in coverage, with some areas yet to adopt this practice [3]. Unfortunately, in the case being discussed, the patient did not undergo such screening.

The significance of early diagnosis and treatment should not be underestimated in IOPD, given its grave impact on mortality and morbidity. ERT with rhGAA is the only approved therapy for Pompe disease. This revolutionary treatment has been demonstrated to reduce cardiac hypertrophy, improve motor function, and prolong survival for IOPD patients. However, ERT is not a cure, and factors like the age of initiation, existing muscle damage extent, presence, or absence of cross-reactive immunologic material (CRIM), and the development of anti-rhGAA antibodies play a pivotal role in determining treatment outcomes. Therefore, early identification of patients who may benefit from ERT is essential [3].

## Conclusions

This case report underscores the importance of genetic counseling, prenatal testing, and early diagnosis in managing Pompe disease. Timely interventions, such as ERT, can improve affected infants’ outcomes and quality of life. Collaborative efforts between healthcare professionals, geneticists, and families are crucial in the fight against this devastating genetic disorder.

## Data Availability

All data generated or analyzed during this study are included in this published article.

## Abbreviations

CRIM: Cross-reactive immunologic material
ERT: Enzyme replacement therapy
GAA: Acid alpha-glucosidase
IOPD: Infantile-onset Pompe disease
LV: Left ventricular
PCR: Polymerase chain reaction
rhGAA: Recombinant human GAA
